# Supplementary material for: Embryonic Trophectoderm Secretomics Reveals Chemotactic Migration and Intercellular Communication of Endometrial and Circulating MSCs in Embryonic Implantation
Source: Int J Mol Sci. 2021 May 26;22(11):5638. doi: 10.3390/ijms22115638 (PMC8199457; doi:10.3390/ijms22115638)
Supplement: Supplementary file 1 [file ijms-22-05638-s001.zip › ijms-1214781 suppl/Methods.pdf]

Sup. Methods 1.

Protein Identification*: *Reverse phase-liquid chromatography-mass spectrometry analysis (RP-LC-MS/MS).

Culture media supplemented with 10% EVs depleted FBS was analyzed to identify possible EVs proteins that remain after EVs depletion to use it as a control. The desalted protein digestion was dried, resuspended in 10 m l of 0.1% formic acid, and analyzed by RP-LC-MS/MS in an Easy-nLC II system coupled to an ion trap LTQ-Orbitrap-Velos-Pro hybrid mass spectrometer (Thermo Scientific). The peptides were concentrated (on-line) by reverse phase chromatography using a 0.1mm × 20 mm C18 RP precolumn (Thermo Scientific) and then separated using a 0.075mm x 250 mm C18 RP column (Thermo Scientific) operating at 0.3 μl/min. Peptides were eluted using a 90min dual gradient. The gradient profile was set as follows: 5–25% solvent B for 68 min, 25–40% solvent B for 22min, 40–100% solvent B for 2min and 100% solvent B for 18 min (Solvent A: 0,1% formic acid in the water, solvent B: 0,1% formic acid, 80% acetonitrile in water). ESI ionization was done using a Nano-bore emitters Stainless Steel ID 30 μm (Proxeon) interface at 2.1 kV spray voltage with S-Lens of 60%. The Orbitrap resolution was set at 30.000. [1]. Peptides were detected in survey scans from 400 to 1600 amu (1 μscan), followed by twenty data-dependent MS/MS scans (Top 20), using an isolation width of 2 u (in mass-to-charge ratio units), normalized collision energy of 35%, and dynamic exclusion applied during 60 seconds periods. The charge-state screening was enabled to reject unassigned and singly charged protonated ions.

Data processing:

Peptide identification from raw data was carried out using the PEAKS Studio X search engine (Bioinformatics Solutions Inc., Waterloo, Ontario, Canada). A database search was performed against UniProt-Bos-taurus.fasta, Reference Proteome (decoy-fusion database). The following constraints were used for the searches: tryptic cleavage after Arg and Lys (semispecific), up to two missed

cleavage sites, and tolerances of 20 ppm for precursor ions and 0.6 Da for MS/MS fragment ions and the searches were performed allowing optional Met oxidation and Cys carbamidomethylation. False discovery rates (FDR) for peptide spectrum matches (PSM) were limited to 0.01. Only those proteins with at least two distinct peptides and at least one unique peptide being discovered from LC/MS/MS analyses were considered reliably identified [2–4].

Protein quantification: by LC-MS/M iTRAQ labeling and high pH fractionation. To quantify EVs and soluble proteins from BBT-9 and BBT-18 by *iTRAQ labeling:* The resultant peptide mixture from desalted proteins tryptic digest (30 mg EVs cargo proteins, 100 mg soluble proteins) was labeled using chemicals from the iTRAQ Reagents 4plex Multiplex Kit (Applied Biosystems, MA, USA). Reagents 115 and 116 were used for BBT-9 and BBT-18 EVs samples, and 114 and 117 for BBT-9 and BBT-18 soluble protein samples respectively as described [5]. Briefly, peptides were dissolved in 30 μL of 0.5 M triethylammonium bicarbonate (TEAB), adjusted to pH 8. For labeling, each iTRAQ reagent was dissolved in 70 μl of ethanol and added to the respective peptide mixture, and then incubated at room temperature for one hour. Labeling was stopped by the addition of 0.1% formic acid. Whole supernatants were dried down and the two samples were mixed to obtain the "2plex-labeled mixture". The mixture was analyzed by RP-LC-MS/MS to check the efficiency of the labeling.

High pH fractionation:

Samples were then fractionated using the Pierce High pH Reversed-Phase Peptide Fractionation Kit (Thermo Fisher Scientific, MA, USA) as described with minor modifications. The samples re-swollen in 0.1%TFA and then, loaded onto an equilibrated, high-pH, reversed-phase fractionation spin column. A step gradient of increasing acetonitrile concentrations (5-80%) in a volatile high-pH (Triethylamine (0.1%)) is then applied to the columns to elute bound peptides into nine different fractions collected by centrifugation. The fractions obtained

from high-pH, reversed-phase 2plex-labeled mixture were dried and stored until analysis by mass spectrometry for quantification.

Protein quantification: RP-LC-MS/MS analysis.

EVs and soluble protein from BBT-9 and BBT-18 fractions were resuspended in 10 ml of 0.1% formic acid and analyzed by RP-LC-MS/MS in an Easy-nLC II system coupled to an ion trap LTQ-Orbitrap-Velos-Pro hybrid mass spectrometer (Thermo Scientific). The peptides were concentrated (on-line) by reverse phase chromatography using a 0.1mm × 20 mm C18 RP precolumn (Thermo Scientific) and then separated using a 0.075mm x 250 mm C18 RP column (Thermo Scientific) operating at 0.3 μl/min. Peptides were eluted using a 90-min dual gradient. The gradient profile was set as follows: 5−25% solvent B for 68 min, 25−40% solvent B for 22min, 40−100% solvent B for 2min and 100% solvent B for 18 min (Solvent A: 0,1% formic acid in the water, solvent B: 0,1% formic acid, 80% acetonitrile in water). ESI ionization was done using a Nano-bore emitters Stainless Steel ID 30 μm (Proxeon) interface at 2.1 kV spray voltage with S-Lens of 60%. The instrument method consisted of a data-dependent top-20 experiment with an Orbitrap MS1 scan at a resolution (m/Δm) of 30,000 followed by either twenty high energy collision dissociation (HCD) MS/MS mass-analyzed in the Orbitrap at 7,500 (Δm/m) resolution. MS2 experiments were performed using HCD to generate high resolution and high mass accuracy MS2 spectra. The minimum MS signal for triggering MS/MS was set to 500. The lock mass option was enabled for both MS and MS/MS mode and the poly-dimethyl cyclosiloxane ions (protonated (Si(CH3)2O))6; m/z 445.120025) were used for internal recalibration of the mass spectra. Peptides were detected in survey scans from 400 to 1600 amu (1 μscan) using an isolation width of 1.3 u (in mass-to-charge ratio units), normalized collision energy of 40% for HCD fragmentation, and dynamic exclusion applied during 60 seconds periods. The charge-state screening was enabled to reject unassigned and singly charged protonated ions.

Protein quantification: Quantitative Data Analysis

Peptide identification from raw data (a single search was performed with all nine raws from the fractionation) was carried out using the PEAKS Studio X search engine (Bioinformatics Solutions Inc., Waterloo, Ontario, Canada). A database search was performed against UniProt-Bos-taurus.fasta, Reference Proteome (decoy-fusion database). The following constraints were used for the searches: tryptic cleavage after Arg and Lys (semispecific), up to two missed cleavage sites, and tolerances of 20 ppm for precursor ions and 0.05 Da for MS/MS fragment ions and the searches were performed allowing optional Met oxidation and Cys carbamidomethylation and fixed iTRAQ 4plex reagent labeling at the N-terminus and lysine residues. False discovery rates (FDR) for peptide spectrum matches (PSM) were limited to 0.01. Only those proteins with at least two distinct peptides and at least one unique peptide being discovered from LC/MS/MS analyses were considered reliably identified and sent to be quantified.

Quantitation of iTRAQ labeled peptides was performed with the PEAKS Studio X search engine, selected "Reporter Ion Quantification iTRAQ" under the "Quantifications" options. We use an auto normalization model that calculates a global ratio from the total intensity of all labels in all quantifiable peptides. The -10LgP, Quality (12), and Reporter Ion Intensity (1e5) were used for Spectrum filter, and Significance (20, PEAKSQ method) was used for peptide and protein abundance calculation. For the protein quantification, we consider protein groups for peptide uniqueness, use unique peptides for protein quantification, and the modified peptides were excluded.

References

1. Alonso, R.; Pisa, D.; Marina, A.I.; Morato, E.; Rábano, A.; Rodal, I., Carrasco, L. Evidence for fungal infection in cerebrospinal fluid and brain tissue from patients with amyotrophic lateral sclerosis. *Int J Biol Sci.* **2015**, 11, 546-558. https://doi.org/10.7150/ijbs.11084
2. Tran, N.H.; Qiao, R.; Xin, L.; Chen, X.; Liu, C.; Zhang, X.; Shan, B.; Ghodsi, A., Li, M. Deep learning enables de novo peptide sequencing from data-independent-acquisition mass spectrometry. *Nat Methods.* **2019**, 16, 63-66. https://doi.org/10.1038/s41592-018-0260-3
3. Tran, N.H.; Zhang, X.; Xin, L.; Shan, B., Li, M. De novo peptide sequencing by deep learning. *Proc Natl Acad Sci U S A.* **2017**, 114, 8247-8252. https://doi.org/10.1073/pnas.1705691114

4. Tran, N.H.; Rahman, M.Z.; He, L.; Xin, L.; Shan, B., Li, M. Complete De Novo Assembly of Monoclonal Antibody Sequences. *Sci Rep*. **2016**, 6, 31730. https://doi.org/10.1038/srep31730
5. Zou, Y. et al. Quantitative iTRAQ-based proteomic analysis of piperine protected cerebral ischemia/reperfusion injury in rat brain. *Neurochem Int*. **2019**, 124, 51-61. https://doi.org/10.1016/j.neuint.2018.12.010
