# Supplementary figures and images for: Embryonic Trophectoderm Secretomics Reveals Chemotactic Migration and Intercellular Communication of Endometrial and Circulating MSCs in Embryonic Implantation
Source: Int J Mol Sci. 2021 May 26;22(11):5638. doi: 10.3390/ijms22115638 (PMC8199457; doi:10.3390/ijms22115638)

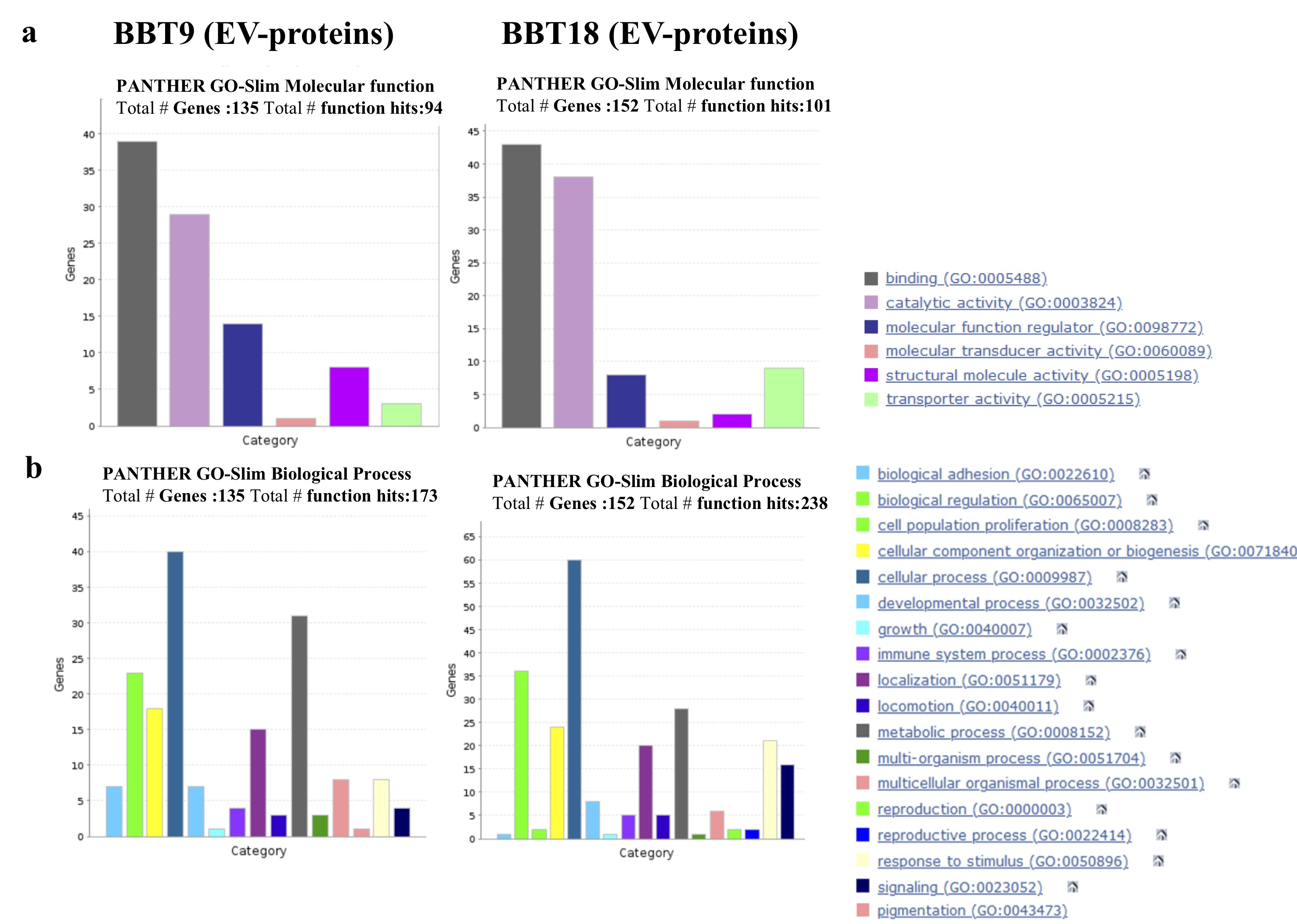

Supplement: Supplementary file 1 [file ijms-22-05638-s001.zip › ijms-1214781 suppl/Figure S1.jpg]

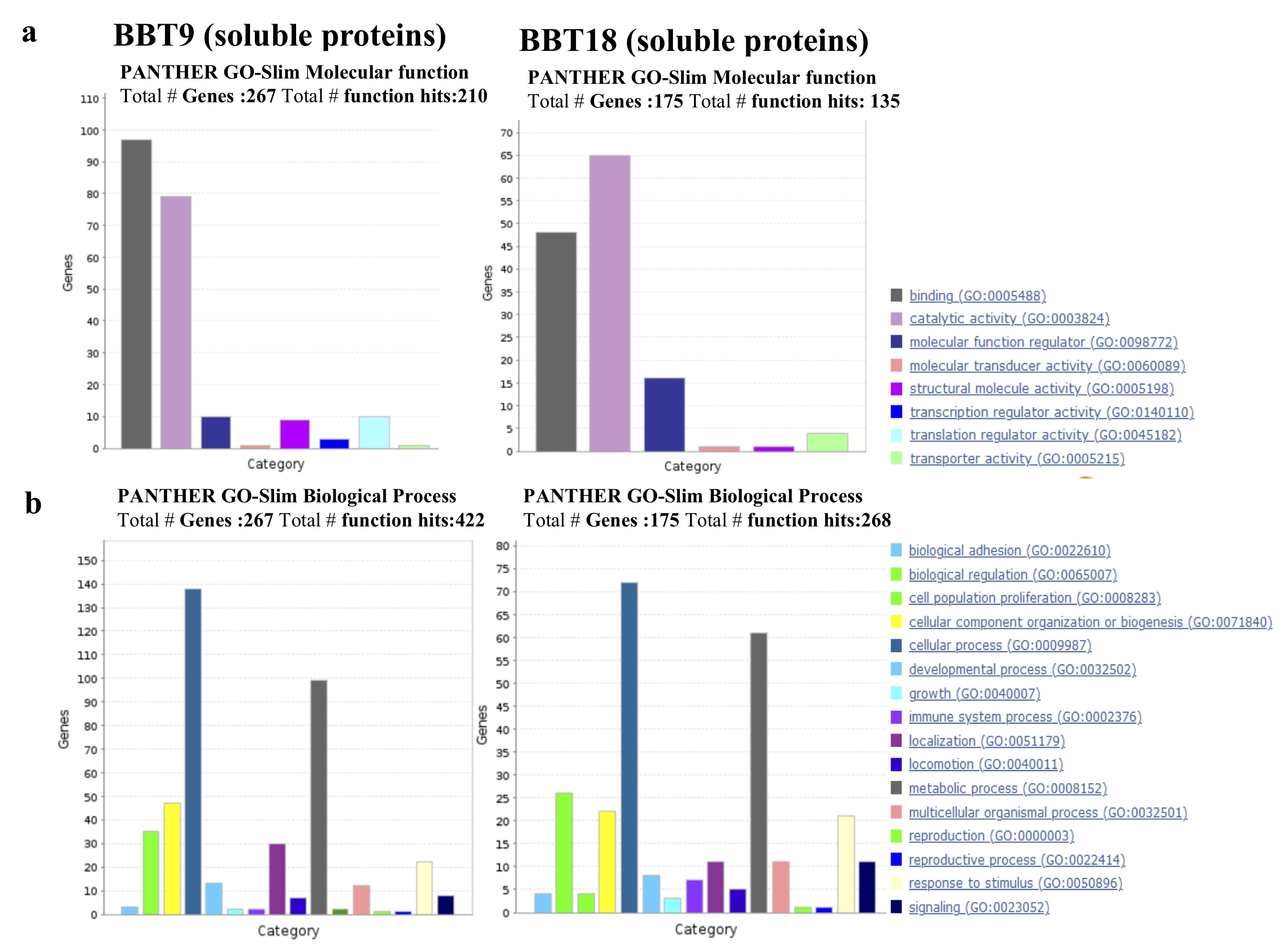

Supplement: Supplementary file 1 [file ijms-22-05638-s001.zip › ijms-1214781 suppl/Figure S2.jpg]

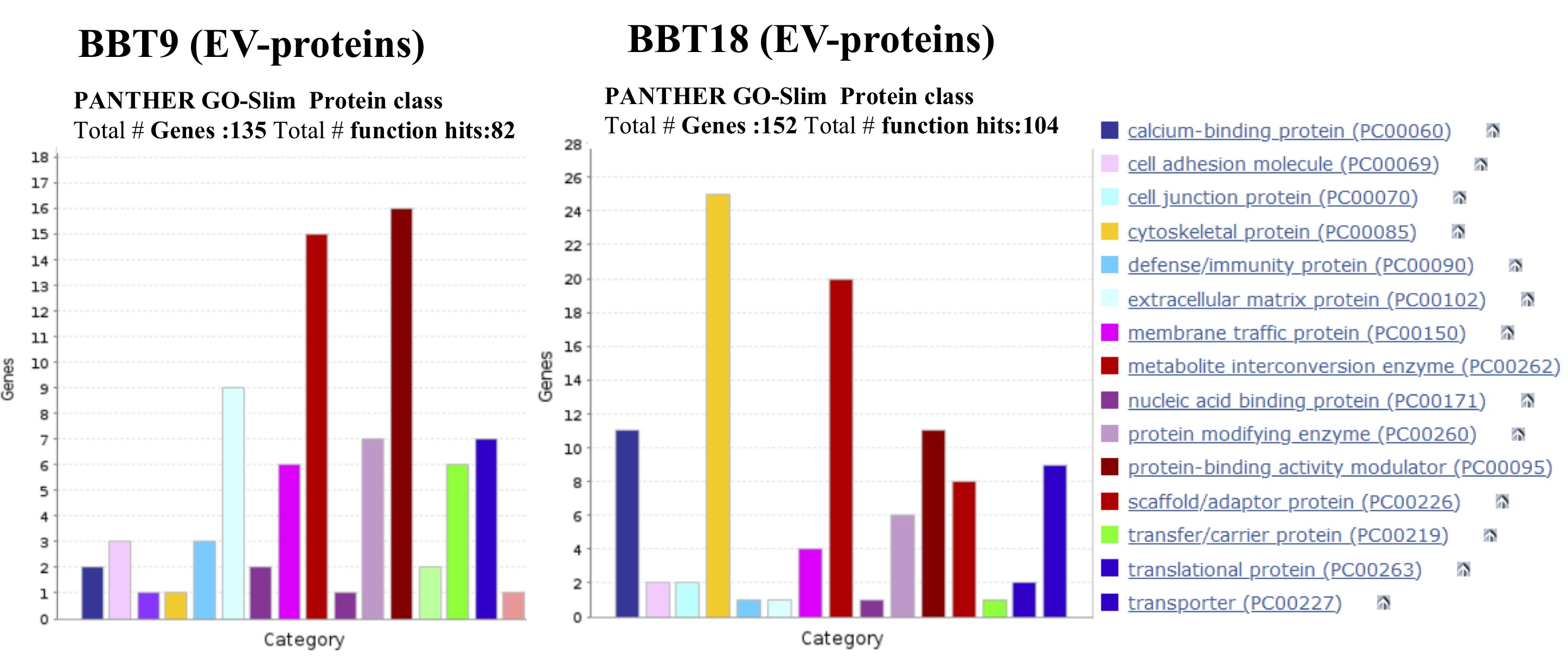

Supplement: Supplementary file 1 [file ijms-22-05638-s001.zip › ijms-1214781 suppl/Figure S3.jpg]

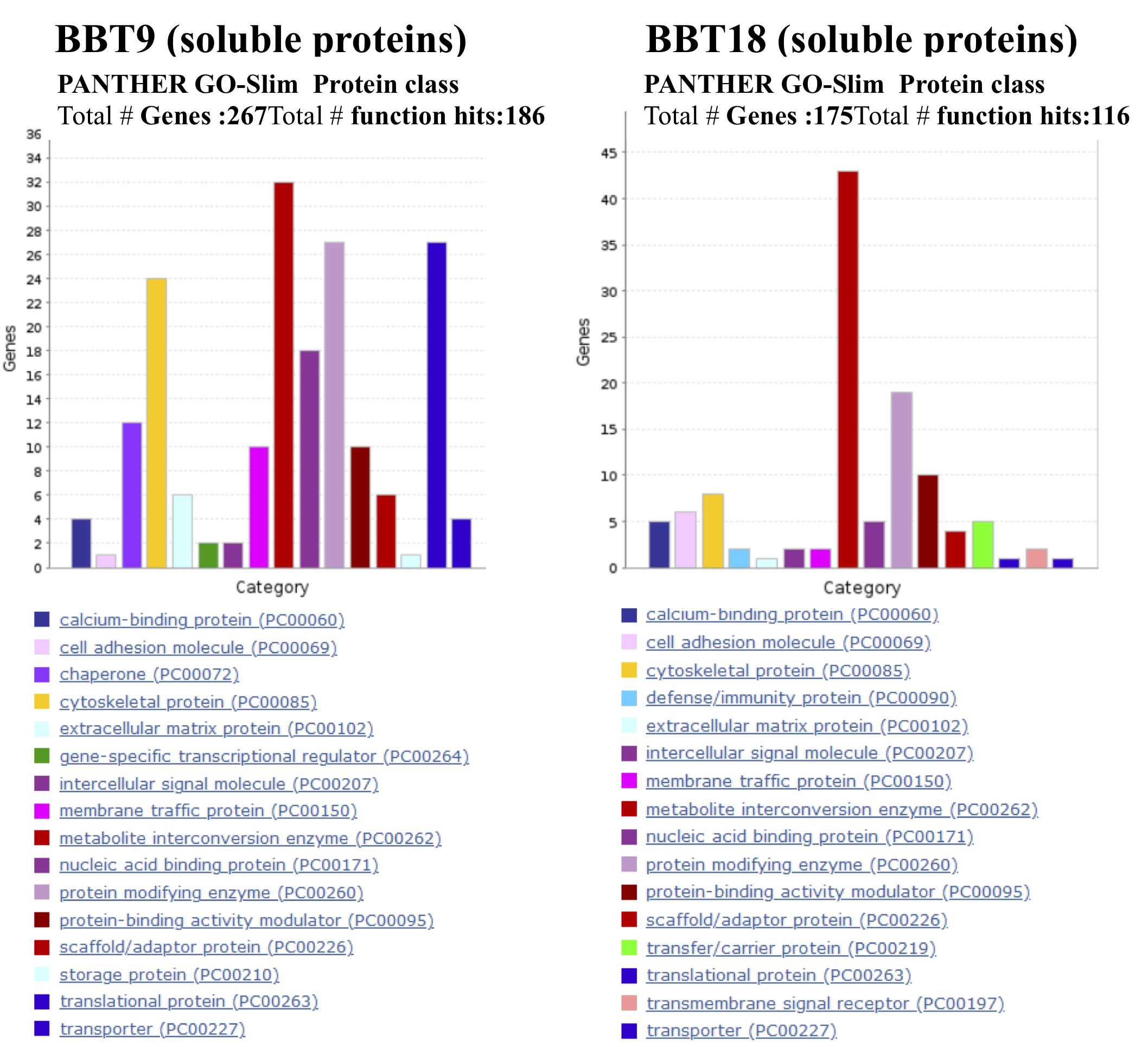

Supplement: Supplementary file 1 [file ijms-22-05638-s001.zip › ijms-1214781 suppl/Figure S4.jpg]

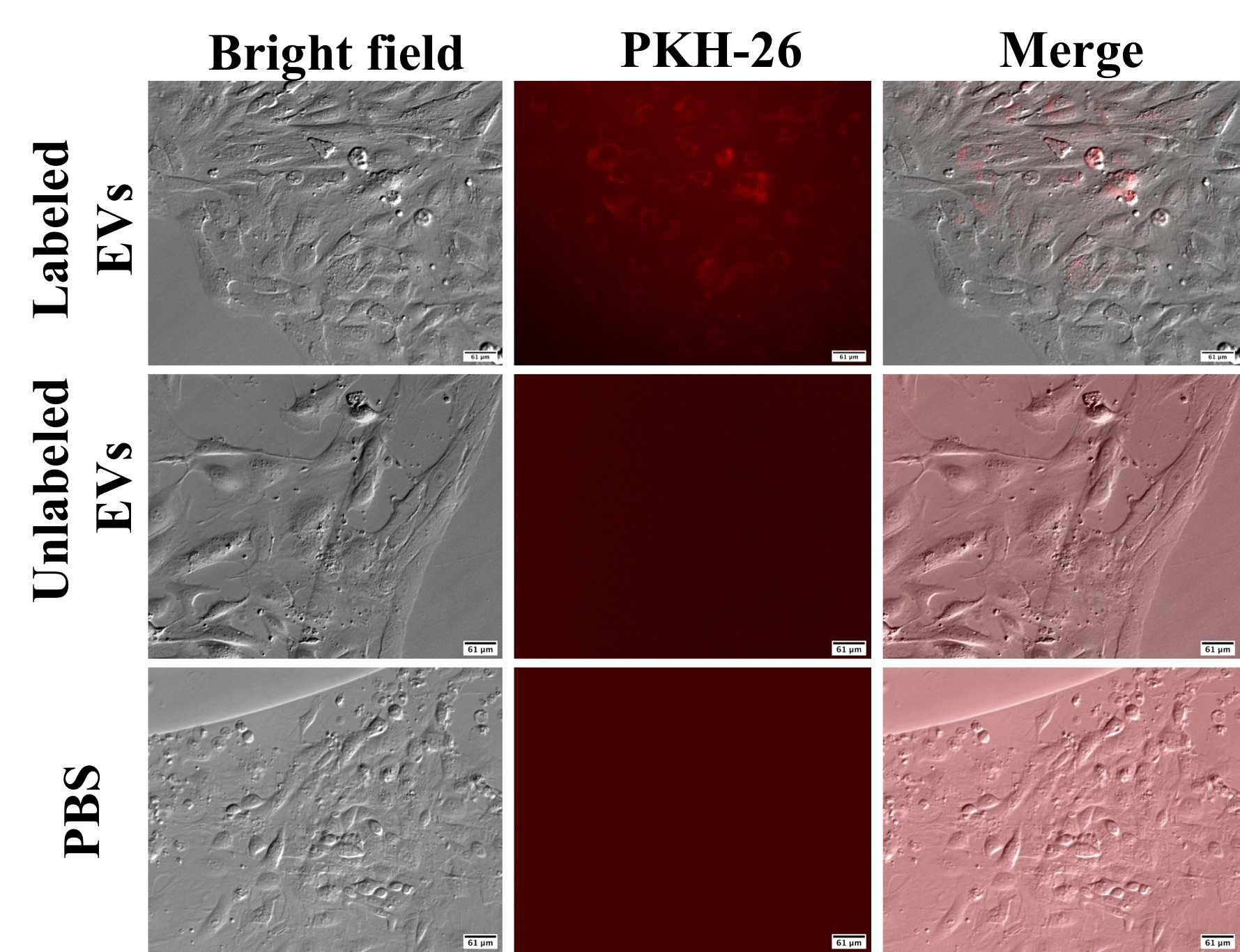

Supplement: Supplementary file 1 [file ijms-22-05638-s001.zip › ijms-1214781 suppl/Figure S5.jpg]
